# The Global Health System: Strengthening National Health Systems as the Next Step for Global Progress

**DOI:** 10.1371/journal.pmed.1000089

**Published:** 2010-01-12

**Authors:** Julio Frenk

**Affiliations:** Harvard School of Public Health, Boston, Massachusetts, United States of America

## Abstract

In the second in a series of articles on the changing nature of global health institutions, Julio Frenk offers a framework to better understand national health systems and their role in global health.

*This is the second in a series of four articles that highlight the changing nature of global health institutions.*

Three circumstances make the present moment unique for global health. First, health has been increasingly recognized as a key element of sustainable economic development [1], global security, effective governance, and human rights promotion [2]. Second, due to the growing perceived importance of health, unprecedented—albeit still insufficient—sums of funds are flowing into this sector [3]. Third, there is a burst of new initiatives coming forth to strengthen national health systems as the core of the global health system and a fundamental strategy to achieve the health-related Millennium Development Goals.

In order to realize the opportunities offered by the conjunction of these unique circumstances, it is essential to have a clear conception of national health systems that may guide further progress in global health. To that effect, the first part of this Policy Forum examines some common misconceptions about health systems. Part two explains a framework to better understand this complex field. Finally, I offer a list of suggestions on how to improve national health system performance and what role global actors can play.

## The Health System: Neither Black Box Nor Black Hole

The increasing interest in national health systems signals a positive shift. As funding for global health has grown during the past years, it has become increasingly clear that this is a necessary but not sufficient condition for progress. Resources should also be used effectively to produce the expected results. In a virtuous circle, those results will help to maintain the momentum of increased funding for health.

Achieving results is precisely what defines health system performance. So if we are to advocate for greater resources, we also need to improve our understanding of these systems. Three common misconceptions are particularly prevalent, which see the health system as a black box, as a black hole, or as a laundry list.

The “black box” misconception is the belief that things are too complicated and we do not understand the intricate mechanisms of health systems, so we must simply get technologies and other inputs in place and then outputs will somehow work their way. Yet we have built a sufficient body of knowledge to be able to open the black box and devise specific interventions to improve the performance of the health system. There is a mounting body of evidence on what works and what doesn't in different settings.

The “black hole” misconception is the common view that no amount of money will suffice to achieve the desired results. As with the dreaded astronomical bodies, health systems absorb enormous amount of energy, but no light comes out of them. Yet, we know that some systems are much more efficient in achieving better results with limited resources.

Finally, the “laundry list” view is a sort of “inventory” approach, in which the health system is defined as a mere list of the different organizations or persons that participate in producing health services, without requiring that such components be coordinated or integrated.

## Expanding Our View on Health Systems

Part of the problem with the health systems debate is that too often it has adopted a reductionist perspective that ignores important aspects. Developing a more comprehensive view requires that we expand our thinking in four main directions.

First, we should think of the health system not only in terms of its component elements (like human resources, financing, hospitals, clinics, technologies, etc.) but most importantly in terms of their interrelations. Second, we should include not only the institutional or supply side of the health system, but also the population. In a dynamic view, the population is not an external beneficiary of the system; it is an essential part of it. This is because, when it comes to health, persons play five different roles: (i) as patients, with specific needs requiring care; (ii) as consumers, with expectations about the way in which they will be treated; (iii) as taxpayers and therefore as the ultimate source of financing; (iv) as citizens who may demand access to care as a right; and most importantly, (v) as co-producers of health through care seeking, compliance with prescriptions, and behaviors that may promote or harm one's own health or the health of others. The importance of this perspective is that it opens the door to population-side interventions to improve the health system, as evidenced by the successful experiences in Mexico and elsewhere with conditional cash transfers that provide incentives for health-promoting behaviors and with insurance programs that empower citizens by subsidizing their demand for explicit entitlements [4].

A third expansion of our understanding of systems refers to their goals. Typically, we have limited the discussion to the goal of improving health. This is, indeed, the defining goal of a health system. However, we must look not only at the level of health, but also at its distribution, which gives equity a central place in assessing a health system. In addition, we must also include other goals that are intrinsically valued beyond the improvement of health. One of those goals is to enhance the responsiveness of the health system to the legitimate expectations of the population for care that respects the dignity of persons and promotes their satisfaction. The other goal is fair financing, so that the burden of supporting the system is distributed in an equitable manner and families are protected from the financial consequences of disease.

Finally, we should expand our view with respect to the functions that a health system must perform. Most global initiatives have been concerned mainly with one of those functions, namely, the direct provision of services, whether they are medical or public health services. This is, of course, an essential function, but for it to happen at all, health systems must perform other enabling functions, such as stewardship, financing, and resource generation, including what is probably the most complex of all challenges, the health workforce.

The four directions I have just summarized form a framework [5] to expand our understanding of health systems so that we may improve them. Specifying the goals allows us to assess the performance of a health system by measuring how well each of the goals is achieved, given the level of health expenditure and the social determinants of health, as measured by indicators like income per capita or educational level. In turn, analysis of the way the functions are carried out enables us to explain variations in performance.

## A LIST for Health Systems Improvement

Actually, we know that there are wide variations in performance by different health systems, even at the same level of income per capita and health expenditure. These variations are due to the influence of several determinants enclosed in the acronym LIST, which stands for leadership, institutions, systems design, and technologies. These determinants are enumerated in decreasing order of complexity, from the bottom up.

### Technologies

No health system can succeed if it does not deliver the appropriate set of interventions, along with their accompanying technologies. This is the aspect that has been better studied and where we have substantial consensus on priorities. Most of the recent increases in global-level support for countries has been directed to expanding the supply of drugs, vaccines, bed nets, and other technologies. However, to work at all these technologies must be embedded in the second element.

### Systems Design

Quality services can only be delivered if a set of systems or subsystems (such as procurement, information, personnel, etc.) are designed so that the required structures and procedures can assure the timely conjunction of human, financial, technological, and knowledge resources. One positive aspect of the recent global initiatives on health systems strengthening is that they address many of these crucial issues. But it is also necessary to take the next step in our acronym.

### Institutions

Development is only possible through the patient construction of institutions, which represent the vehicles whereby human beings mobilize their talents, values, and interests towards the pursuit of shared goals. Institutions also introduce certainty to transactions and articulate incentives. A crucial institution is the ministry of health. Despite its central importance for the stewardship function, many countries are far from having an optimal ministry of health. In this regard, there is a sharp contrast with ministries of finance, where the imperatives of globalization have imposed a fairly homogeneous level of technical proficiency across countries. Ministries of health should certainly be sensitive to local realities, but there is a technical core that is increasingly connected to global networks and should therefore be strengthened through global efforts. Institution building is always tough because it requires long-term investments that are often obliterated by short-term political pressures. This problem is related to the last element in the LIST.

### Leadership

Probably the most complex challenge in health systems is to nurture persons who can develop the strategic vision, technical knowledge, political skills, and ethical orientation to lead the complex processes of policy formulation and implementation. Without leaders, even the best designed systems will fail.

## Knowledge and Action

The present moment offers a unique opportunity to advance specific proposals on each of the four elements of health systems strengthening: greater access to life-saving technologies, improvements in critical subsystems, long-term investments in institution building, and leadership development. However, for these investments to be successful, they must be linked to concrete health outcomes. In this respect, global health requires a new way of thinking and acting in order to bridge the traditional divide between the “vertical” approach, focusing on technical interventions for specific disease priorities [6], and the “horizontal” approach, aimed at strengthening the overall structure and functions of the health system but without a clear sense of priorities. The solution is a truly “diagonal” approach, whereby explicit intervention priorities are used to drive improvements of the health system [7].

Health systems are the main instrumentality to close the knowledge–action gap. To realize this potential, it will be necessary to mobilize the power of evidence to promote change. Yet all too often reform efforts are not evaluated adequately. Each innovation in health systems constitutes a learning opportunity. Not to take advantage of these opportunities condemns us to rediscover at great cost what is already known or to repeat past mistakes. For this reason, the current surge of initiatives on health systems strengthening must be accompanied, from the outset, by an effort to generate a process of shared learning among countries. There is an urgent need to build up a body of knowledge on what works and what does not, so that each country is better equipped to adopt and adapt the lessons learned from every other nation. Shared learning would be greatly assisted by a global repository of evidence on health system performance [8].

This type of evidence is a global public good. Therefore, its funding and coordination requires international action, with far more attention than it has received so far. It also requires a common framework for monitoring and evaluation of interventions that promotes comparability of data, transparency of methods, and accountability to the global community. In this way, knowledge and action will reinforce each other, bringing the world closer to the common goal of better health for all.
